# Mechanical Properties of GaN Single Crystals upon C Ion Irradiation: Nanoindentation Analysis

**DOI:** 10.3390/ma15031210

**Published:** 2022-02-05

**Authors:** Zhaohui Dong, Xiuyu Zhang, Shengyuan Peng, Fan Jin, Qiang Wan, Jianming Xue, Xin Yi

**Affiliations:** 1 Department of Mechanics and Engineering Science, College of Engineering, Peking University, Beijing 100871, China; zhdong@pku.edu.cn; 2 State Key Laboratory of Nuclear Physics and Technology, School of Physics, Peking University, Beijing 100871, China; xiuyuzhang@pku.edu.cn (X.Z.); 2101110314@pku.edu.cn (S.P.); 3 Institute of Systems Engineering, China Academy of Engineering Physics, Mianyang 621999, China; jinfan2046@163.com; 4 HEDPS and Center for Applied Physics and Technology, College of Engineering, Peking University, Beijing 100871, China

**Keywords:** GaN single crystals, mechanical properties, ion irradiation, nanoindentation, pop-in, activation volume, dislocation nucleation

## Abstract

Mechanical properties of gallium nitride (GaN) single crystals upon carbon ion irradiation are examined using nanoindentation analysis at room temperature. Pop-in events in the load-depth curves are observed for unirradiated and irradiated GaN samples. A statistical linear relationship between the critical indentation load for the occurrence of the pop-in event and the associated displacement jump is exhibited. Both the slope of linear regression and the measured hardness increase monotonically to the ion fluence, which can be described by logistic equations. Moreover, a linear relationship between the regression slope as a micromechanical characterization and the hardness as a macroscopic mechanical property is constructed. It is also found that the maximum resolved shear stress of the irradiated samples is larger than that of the unirradiated samples, as the dislocation loops are pinned by the irradiation-induced defects. Our results indicate that the nanoindentation pop-in phenomenon combined with a statistical analysis can serve as a characterization method for the mechanical properties of ion-irradiated materials.

## 1. Introduction

Owing to excellent physical and chemical properties, such as high mobility, high thermal conductivity, thermal stability, good heat dissipation [1], gallium nitride (GaN), as a wide bandgap semiconductor material, has become one of the most promising raw materials for electronic devices. To evaluate the function of GaN-based devices in environments of high radiation dose, studies on the radiation effect on the mechanical and physical properties of GaN samples are called for.

With features of high damage efficiency, low cost, and low residual radioactivity, nowadays, ion irradiation serves as a main method for studying the radiation effect on material properties. Irradiation could induce material defects such as vacancies, interstitial atoms, and dislocation loops. These defects in turn cause a certain extent of lattice disorder and stress-field variation. Low-level stress could significantly affect the photoelectric properties of GaN, such as the bandgap, and damage the performance of the device, while high-level stress could cause local plastic deformation of the material, dislocations to increase, and even cracking, resulting in device failure [2,3,4]. So far, extensive studied have been performed on the effects of irradiation dose, incident ion species, irradiation temperature, and annealing conditions on the dynamic process of lattice disorder and defect migration of GaN samples [5,6,7]. In comparison, only a few studies have been carried out to investigate the mechanical properties of GaN single crystals [8,9]. However, the studies on radiation damage and mechanical behaviors of GaN samples are uncoupled, and there are rare studies considering the impact of irradiation on the mechanical properties of GaN samples, especially for GaN single crystals minimizing the effect of grain boundaries on hindering the dislocation motion and trapping irradiation defects [10]. Therefore, studies of the effect of irradiation on the mechanical properties of GaN single crystals are called for, so as to aid the fundamental understanding and engineering evaluation of GaN and GaN-based device performances.

The penetration depth of ion beams is generally limited within a range from tens of nanometers to several micrometers, and the distribution of irradiation-induced defects is non-uniform within the irradiated layer [11]. These aspects make conventional measurement techniques hardly appropriate for determining the mechanical properties of ion-irradiated samples, and small-scale testing approaches are called for. In comparison with small-scale testing methods, such as microcolumn stretching, compression and bending experiments of small samples, the sample preparation for nanoindentation is relatively simple and a large amount of effective experimental data could be obtained in a relatively short period of time, making nanoindentation an effective, convenient, and popular experimental technology for investigating mechanical properties of ion-irradiated samples [11,12,13]. Further studies indicate that the statistical analysis of pop-in events in the load-depth curves of nanoindentation can be used to quantify irradiation damage at a low irradiation dose [13].

In this work, we experimentally investigate the effect of C ion irradiation on the hardness of GaN single crystals using nanoindentation analysis at room temperature. Pop-in events for unirradiated and irradiated GaN samples are analyzed. It is found that the critical indentation load for the occurrence of the pop-in event and the associated displacement jump follows a statistical linear relationship. The hardness increases monotonically to the ion fluence, following logistic equations. A linear relationship between the slope of the critical load–displacement jump regression and the hardness is determined. Our results shed light on the influence of ion irradiation on mechanical properties of GaN single crystals and have implications for developing high-performance wide bandgap semiconductor-based devices.

## 2. Materials and Methods

The samples are freestanding wurtzite-type undoped c-plane (0001) GaN single crystals fabricated using hydride vapor phase epitaxy (HVPE) (Suzhou Nanowin Science and Technology Co., Ltd., Suzhou, China). A detailed overview of the mechanism and features of HVPE for the growth of III–V and nitride semiconductor structures can be found in [14]. The sample size is 10.0 mm × 10.5 mm × 0.35 mm. The density of in-grown dislocations is about 3 × 10^6^ cm^−2^, determined by cathodoluminescence imaging. The crystal orientation is identified using X-ray diffraction. The Ga surface is treated using chemical mechanical polishing with colloidal silica nanoparticles, achieving a mirror-like surface with surface roughness of less than 0.2 nm.

**Figure 1:**
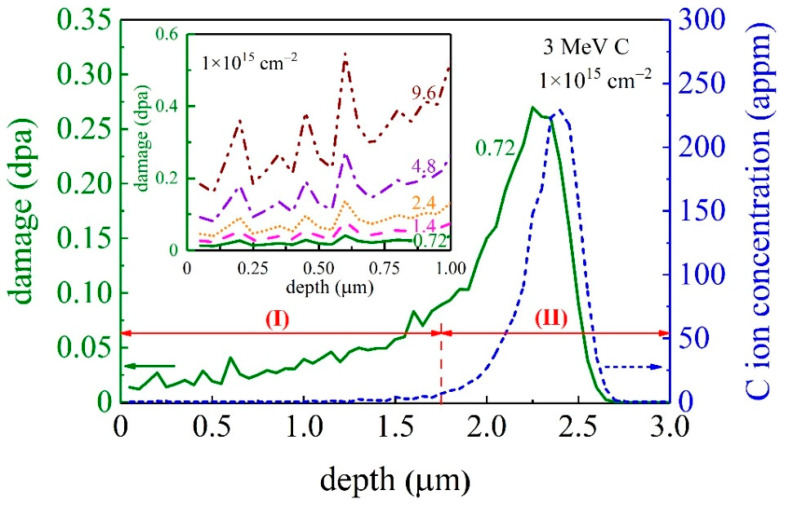
Depth profiles of the displacement damage and C^+^ ion concentration of GaN single crystals implanted with C^+^ at 3 MeV with an ion fluence of 0.72 × 10^15^ cm^−2^. Inset is a magnified view of the damage-depth region at all five ion fluences from 0.72 × 10^15^ cm^−2^ to 9.6 × 10^15^ cm^−2^.

The GaN single crystal samples are irradiated with 3 MeV C^+^ ions with different fluences of 0.72 × 10^15^ ions/cm^2^, 1.4 × 10^15^ ions/cm^2^, 2.4 × 10^15^ ions/cm^2^, 4.8 × 10^15^ ions/cm^2^, and 9.6 × 10^15^ ions/cm^2^. During ion irradiation, the ambient temperature is room temperature (23 °C). The flux of the C^+^ ion is set at 1 × 10^12^ ions/(cm^2^·s) to avoid the increase in sample temperature. The displacements-per-atom (dpa) values of energetic ions are calculated using SRIM-2013 [15] with “Quick” Kinchin-Pease option [16]. The depth profiles of dpa and ion concentration are shown in Figure 1. According to the dpa profile, the damaged layer can be roughly divided into two regions. Region I, a near surface plat region in which the dpa value gradually increases with depth, and region II, a heavily damaged region with a significantly non-uniform damage distribution. In this study, the dpa value refers to the average dpa value over region I. The thickness of region I is around 1.75 μm.

To study the effects of C^+^ ion irradiation on the mechanical properties of GaN single crystals, a nanoindentation analysis using Hysitron TI 980 Triboindenter (Bruker, Minneapolis, MN, USA) system is conducted. The diamond Berkovich indenter is used and its effective tip radius is calibrated as *R* = 351 nm using standard techniques [17,18]. The projected contact area of the tip is calibrated and the frame stiffness is corrected by indenting fused quartz. The indentation test procedure is load controlled, adopting a trapezoidal load-time input function with a 5 s linear loading segment, a 2 s holding segment at peak load, and a 5 s linear unloading segment. The holding segment reduces the effect of creep [19]. To obtain the sample hardness, at least 20 indents are performed on each sample with the maximum load of 10 mN. To observe and analyze the pop-in effect more precisely, at least 200 indents are performed on each sample with a maximum load of 2 mN. The indentation load at which the pop-in event occurs and the pop-in depth or called displacement excursion are recorded. Neighboring indentation sites of a given sample have sufficient separation distance to avoid possible interference. All nanoindentation tests are performed at room temperature (23 °C).

## 3. Results and Discussion

**Figure 2:**
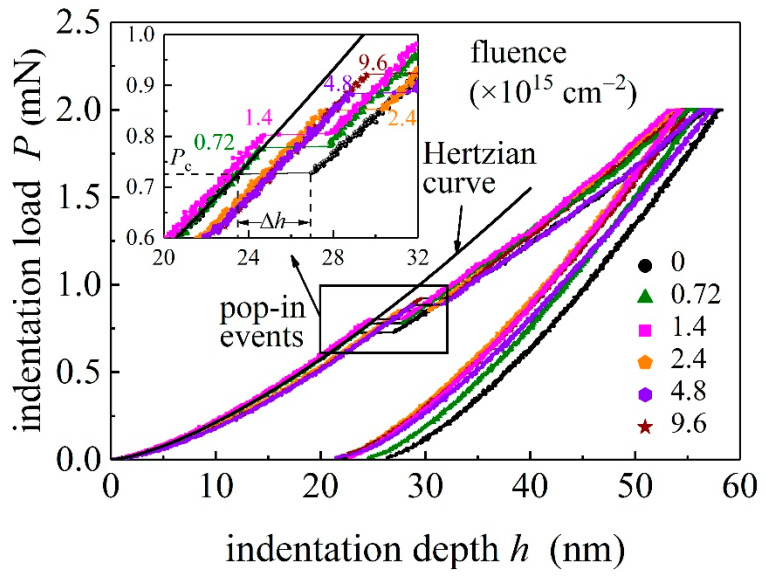
Load–depth (*P*-*h*) curves for GaN single crystals obtained from the nanoindentation experiments with maximum load of 2 mN. The GaN samples are subjected to different values of ion fluence *D* from 0.72 × 10^15^ cm^−2^ to 9.6 × 10^15^ cm^−2^. The inset shows pop-in events in the loading parts of the *P*-*h* curves.

Figure 2 presents the representative load-depth (*P*-*h*) curves for GaN single crystals subjected to different ion fluences. The maximum load is 2 mN. The maximum indentation depth is less than 10% of the thickness of region I around 1.75 μm, indicating that the substrate effect on the indentation could be neglected [17]. Regardless of the irradiation fluence values, pop-in events during loading are observed. Similar pop-in events on unirradiated GaN samples have been observed [8,9,20]. The indentation load at which the first pop-in occurs is referred to as the critical load *P*_c_, and the corresponding displacement jump ∆*h* (length of the horizontal plateau) is referred to as the displacement excursion. For displacement-controlled nanoindentation experiments, a vertical load drop could be observed on the load-depth curve with a pop-in.

According to the Hertzian elastic contact theory [21], the elastic response of a sample to a spherical indentation obeys

(1) $$P=\frac{4}{3}E^{*}R^{1/2}h^{3/2},$$

where *P* is the applied indenter load, *R* is the radius of the indenter tip, *h* is the indentation depth, and the reduced modulus E* combines the moduli of the indenter and sample as

$$\frac{1}{E^{*}}=\frac{1-\nu_{i}^{2}}{E_{i}}+\frac{1-\nu_{\mathrm{GaN}}^{2}}{E_{\mathrm{GaN}}}$$

with *ν* and *E* denoting the Poisson ratio and Young’s modulus, respectively, and subscripts i and GaN are used to identify quantities pertaining to the indenter tip and GaN sample, respectively. As indicated in Figure 2, the Hertzian Equation (1) fits well with the initial portion of the load–depth curve before pop-in, indicating a perfect elastic response at *P* < *P*_c_ (for clarity only the fitting for the unirradiated case is shown here). As *P* exceeds *P*_c_, the *P*-*h* curves deviate from the Hertzian equation with not only a horizontal shift but also a different curve slope, indicating that the sample deformation has transformed into an elastic-plastic way. The load *P*_c_ is regarded as a critical load for the onset plasticity. In comparison to unirradiated GaN samples, the pop-in phenomenon for ion-irradiated GaN samples is associated with a larger critical load.

The occurrence of pop-in events is stochastic. As indicated in Table 1, there is no evident law for the distribution of the critical load *P*_c_ with the ion fluence *D*. However, it has been reported in ref. [22] that the critical load *P*_c_ and the displacement excursion ∆*h* increase after irradiation. In contrast, some studies report that the critical load decrease after irradiation [23]. In addition, existing studies show that the presence of impurity atoms could increase the critical load for pop-in [24,25,26]. These contradictions may be attributed to the different interaction between dislocation and irradiation.

To correlate the pop-in behavior to the irradiation-induced defects, the critical load *P*_c_ is converted to the maximum resolved shear stress *τ*_max_ as [21]

$$\tau_{\max}=0.31{\left(\frac{6P_{c}E^{*2}}{\pi^{3}R^{2}}\right)}^{1/3}$$

The range and average value of *τ*_max_ for unirradiated and irradiated samples are listed in Table 1. The theoretical shear strength of GaN is 23.76 GPa [27], and *τ*_max_ of unirradiated and irradiated samples does not exceed 67% of the theoretical shear strength of GaN. This implies that the onset of pop-in events might be triggered by the presence of dislocations that contribute to the heterogeneous dislocation nucleation of indentation-induced slip during loading [28]. In addition, the *τ*_max_ of irradiated samples is larger than that of the unirradiated samples, because the pre-existing dislocation loops before indentation are pinned by the irradiation-induced defects, and the extent of the increase in *τ*_max_ is related to the pinning extent of the defects [22,28].

**Figure 3:**
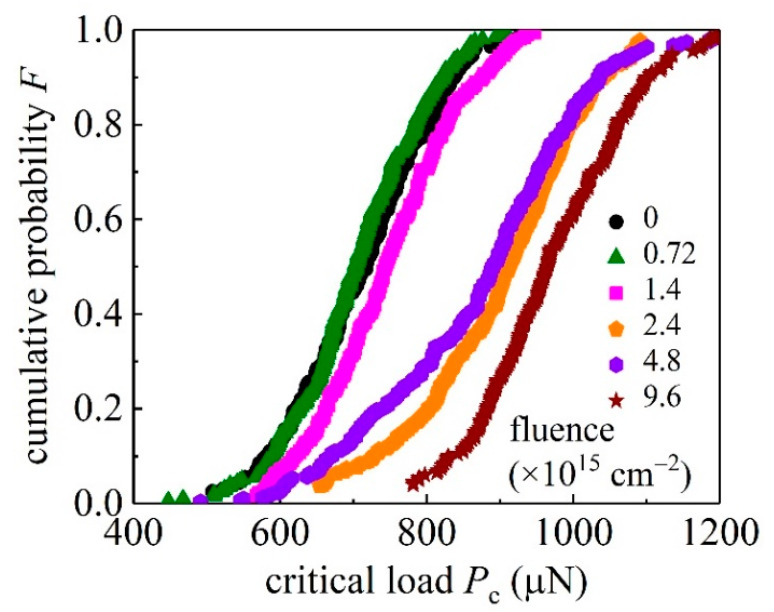
Cumulative pop-in probability as functions of the critical load *P*_c_ at different values of ion fluence *D*.

To describe the stochastic feature of pop-in events, a quantity named the cumulative probability, the probability that a pop-in event occurs before the critical load reaches a value *P*_c_, has been introduced [29,30]. Figure 3 presents the cumulative pop-in probability *F* as functions of *P*_c_ at different values of ion fluence *D*.

According to the statistical method proposed by Schuh et al. [29,30], the cumulative probability *F* can be correlated with the critical load *P*_c_ of pop-in *F* = *F*(*P*_c_) as

(2) $$\ln[-\ln(1-F)]=\alpha P_{c}^{1/3}+\beta (P_{c}),$$

where *β*(*P*_c_) is a function of weak dependency on *P*_c_. The parameter *α* in Equation (2) is correlated with the activation volume *V* through

(3) $$V=\frac{\pi }{0.47}{\left(\frac{3R}{4E^{*}}\right)}^{2/3}k_{B}T\cdot \alpha$$

**Figure 4:**
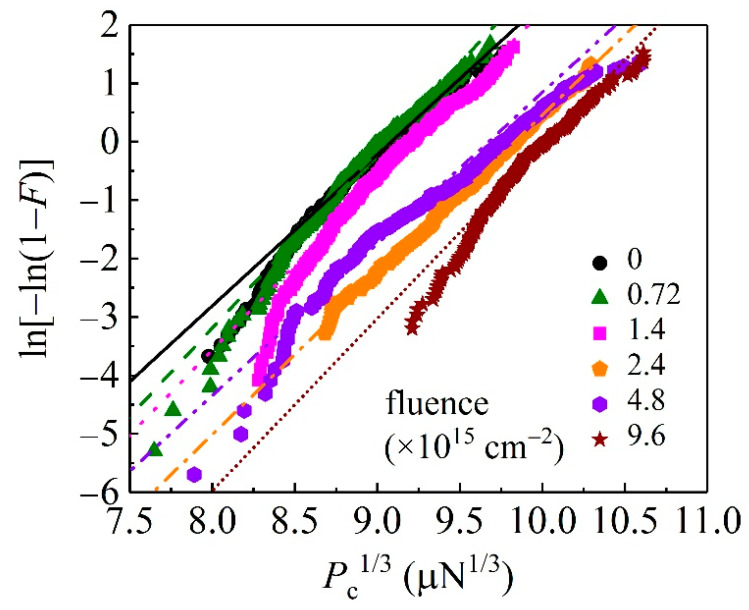
Extracting activation volume from experimental data using Equation (2). Straight lines are best linear fits of Equation (2) to the symbols.

Plotting ln[−ln(1 − *F*)] versus *P*^1/3^ in Figure 4, one can see that experimental data roughly fall onto linear lines, consistent with Equation (2). Slopes of these fitted straight lines determine values of *α* for unirradiated and irradiated GaN samples, from which values of the activation volume *V* can be obtained using Equation (3). For GaN single crystals, the magnitude of the Burgers vector **b** = 1/3[2−1−10] is 3.191 Å [27]. The value of *V* varies as the ion fluence varies (see Table 1), implying heterogeneous dislocation nucleation during the pop-in at different ion fluence [31].

**Figure 5:**
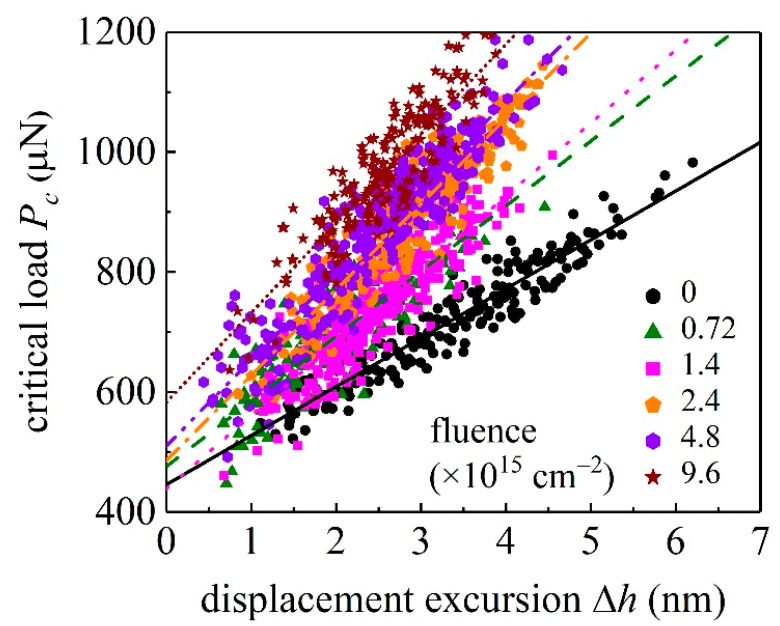
The critical load for pop-in versus the corresponding displacement excursion for unirradiated and irradiated GaN samples. Straight lines are linear fits to experimental pop-in data (symbols).

**Figure 6:**
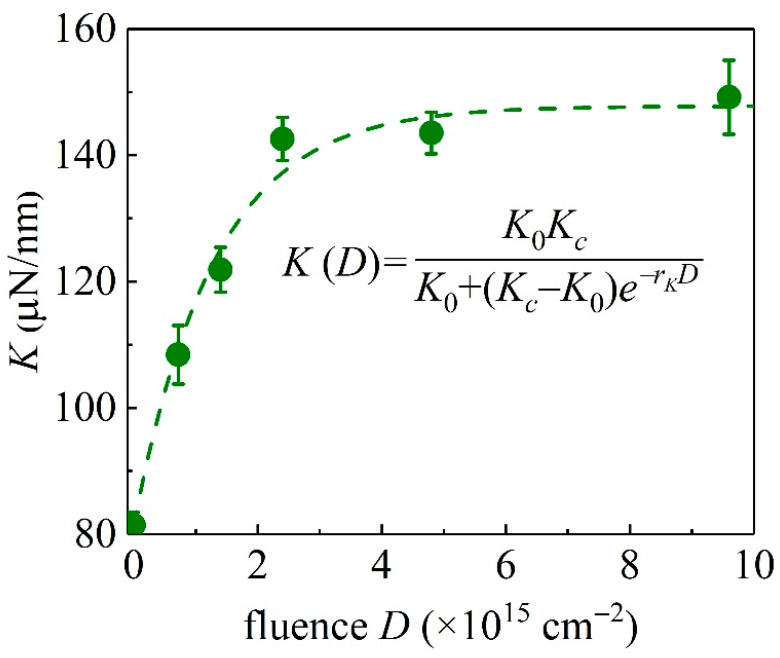
*K* values of GaN single crystal samples as a function of ion fluence *D*.

In indentation experiments, the pop-in event is caused by the transformation of the elastic deformation to plastic deformation [32]. Figure 5 plots the critical load *P*_c_ versus ∆*h*, at different values of ion fluence *D*. At least 200 indentation experiments of unirradiated and irradiated GaN samples have been performed. It can be seen that the pop-in event does not necessarily occur at the same critical load, and a statistical linear dependency of *P*_c_ on ∆*h* is exhibited. The larger the ion fluence *D* is, the larger the slope *K* = d*P*_c_/d(∆*h*) is. A similar linear relationship between *P*_c_ and ∆*h* for unirradiated GaN sample has been observed for unirradiated GaN samples [33]. Values of *K* at different values of *D* are shown in Table 1 and plotted in Figure 6. It is shown that the *K*-*D* relationship can be described by the governing logistic equation

(4) $$\frac{dK}{dD}=r_{K}K\left(1-\frac{K}{K_{c}}\right)\;\mathrm{with}\;K(0)=K_{0}$$

Here, *r_K_* is the increment rate, *K*_c_ is the capacity of the *K* value, and *K*_0_ is the value of *K* at zero ion fluence (*D* = 0). From Equation (4), one has

(5) $$K(D)=\frac{K_{0}K_{c}}{K_{0}+(K_{c}-K_{0})e^{-r_{K}D}},$$

where *r_K_* = 1.09599 × 10^−15^ cm^2^, *K*_c_ = 146.62116 μN/nm, and *K*_0_ = 81.35841 μN/nm are obtained with regression to the experimental data in Figure 6 with 10^15^ cm^−2^ and μN/nm taken as the unit of fluence and *K*, respectively.

**Figure 7:**
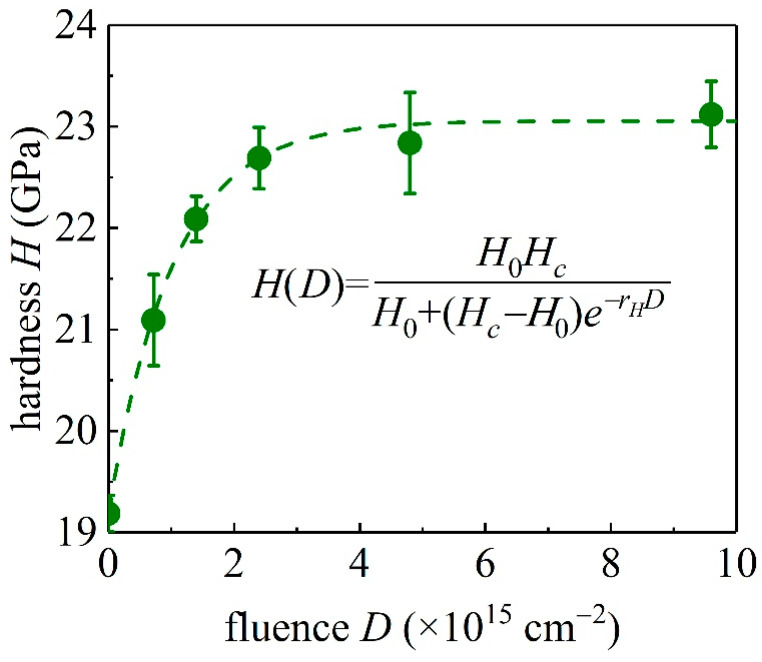
The hardness *H* of GaN single crystal samples as a function of ion fluence *D*.

Following the well-established Oliver and Pharr method [17], one can determine the hardness of the unirradiated and irradiated GaN samples from the nanoindentation experiments. Here, we extract the value of *H* from *P*-*h* curves measured at a maximum load of 10 mN. In these experiments, the indention depth does not exceed 165 nm, less than 1/10 of the thickness of region I in Figure 1. Therefore, the substrate effect on the measured hardness is negligible. The Poisson ratio *ν*_GaN_ = 0.22 is adopted here [34]. The Young’s modulus and Poisson ratio of the diamond indenter are *E*_i_ = 1141 GPa and *ν*_i_ = 0.07, respectively. Each sample is measured with more than 20 indentations to obtain an average value. As indicated in Figure 7 and similar to the analysis of Figure 6, the hardness *H* of the GaN single crystal increases monotonically to the ion fluence *D* and their relation can be described by a logistic equation

(6) $$\frac{dH}{dD}=r_{H}H\left(1-\frac{H}{H_{c}}\right)\;\mathrm{with}\;H(0)=H_{0}$$

Here, *r_H_* is the increment rate, $H_{c}$ is the hardness capacity, and *H*_0_ is the hardness for unirradiated samples. From equation (6), one has

(7) $$H(D)=\frac{H_{0}H_{c}}{H_{0}+(H_{c}-H_{0})e^{-r_{H}D}},$$

where *r_H_* = 1.09008 × 10^−15^ cm^2^, *H*_c_ = 23.03968 GPa, and *H*_0_ = 19.18533 GPa are the fitting parameters with 10^15^ cm^−2^ as the unit of fluence *D* and GPa as the unit of hardness *H*. The obtained mechanical parameters of unirradiated and irradiated GaN single crystals are summarized in Table 1.

As indicated in Figure 7, the hardness of GaN single crystals is modified by the ion bombardment. For the unirradiated GaN single crystals, the hardness is 19.2 ± 0.2 GPa, consistent with reported values in the literature [8,9]. As the ion influence *D* increases, the hardness *H* of GaN single crystals increases and saturates following Equation (7). Related to the elastoplastic behavior of single crystal materials, the hardness can be regarded as the sum of the resistance of the atomic bonds per unit area under the indenter to the indentation deformation, which is mainly attributed to the bond density, shear strength, and the move of dislocations [35], and these aspects could be tuned by irradiation. On one hand, the irradiation-induced defects gradually reduce the density and strength of chemical bonds, increase the lattice spacing, and cause a decrease in hardness. On the other hand, the irradiation defects interact with the dislocation, inhibit the dislocation motion, and increase the resistance to volume changes, leading to an increase in hardness [36]. Therefore, the irradiation effect on hardness is a result of the competition between structural defects and stress field caused by irradiation.

**Figure 8:**
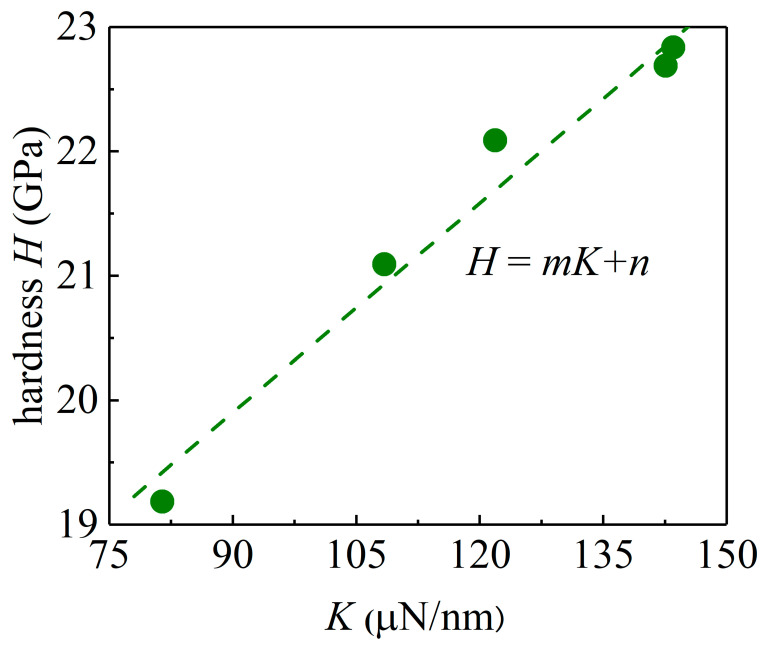
Relationship between the hardness *H* and the *K* values for GaN samples. The dotted straight line is a linear fit of the extracted data.

As indicated in Equations (5) and (7), the *H*-*D* and *K*-*D* relationships can be captured by the logistic equations of the same form, which indicates that there might exist a simple relationship between *H* and *K*. By extracting the values of *H* and *K* and plotting the *H*-*K* relationship in Figure 8, one can observe that the *H*-*K* relationship can be described by

*H* = *mK* + *n*

where *m* = 0.05606 μm^−^^1^ and *n* = 14.86 GPa are the two fitting parameters. Similar linear behaviors have also been observed in the nanoindentation of compound semiconductors [28] and even perovskites such as APbX_3_ (A=Cs, CH_3_NH_3_; X=I, Br) single crystals [37]. Based on a simple nanoindentation model proposed by Field and Swain [32], *K* has also been predicted to have a linear dependency on the hardness *H*. The hardness *H* is a macroscopic mechanical property and the parameter *K* as the slope d*P*_c_/d(∆*h*) characterizing the material micromechanical responses. It is interesting to note that an appropriate choice or introduction of the statistical trend of the material micromechanical responses can be used to characterize the macroscopic mechanical properties. Therefore, the nanoindentation pop-in phenomenon combined with a statistical analysis can be used as a characterization method for the mechanical properties of ion-irradiated materials.

In the present experimental study, we have focused on the nanoindentation of ion-irradiated GaN samples at room temperature. Future work will be aimed at evaluating the effects of neutron- and ion-irradiation damage as well as high temperatures on the mechanical properties of III–V and nitride semiconductors using more sophisticated experimental techniques [38] and theoretical modeling [39].

## 4. Conclusions

Using nanoindentation technique, the effects of C ion irradiation with different ion fluences on the mechanical and physical properties of GaN single crystals are investigated, including the critical load and displacement excursion of pop-in events, maximum resolved shear stress, activation volume, and the hardness. For both unirradiated and irradiated GaN samples upon nanoindentation, the sample deformation is perfectly elastic before the first pop-in event. The mechanism of pop-in events is a heterogeneous dislocation nucleation process. It is found that there is a statistical linear relationship between the critical indentation load, which exhibits a statistical linear dependence on the displacement excursion. As the ion fluence increases, both the slope of that linear regression and the measured hardness increase monotonically to the ion fluence following the logistic feature. Moreover, the maximum shear stress of irradiated GaN samples is larger than that of the unirradiated samples, attributed to pinning dislocation loops by the irradiation-induced defects. Combining nanoindentation pop-in events with statistical analysis could characterize the irradiation effect on the mechanical properties of materials.

## Figures and Tables

**Table 1:** Measured mechanical and physical properties of unirradiated and irradiated GaN single crystals by nanoindentation.

Fluence *D*	Hardness *H*	*τ*_max_	Mean Value of *τ*_max_	*P*_c_	Mean Value of *P*_c_	*V* ^1^	∆*h*	*K*
(10^15^ cm^−2^)	(GPa)	(GPa)	(GPa)	(μN)	(μN)	(Å^3^)	(nm)	(μN/nm)
0	19.2 ± 0.2	11.0–14.5	13.1	423.3−982.4	712.8	7.24 (=0.22*b*^3^)	0.5–6.2	81.5 ± 2.0
0.72	21.1 ± 0.4	11.2–14.2	13.0	447.4–907.9	703.4	8.52 (=0.26*b*^3^)	0.6–4.5	108.4 ± 4.7
1.4	22.1 ± 0.2	11.3–14.6	13.3	461.4–994.4	745.6	8.16 (=0.25*b*^3^)	0.7–4.5	121.9 ± 3.6
2.4	22.6 ± 0.3	11.6–15.8	14.1	501.8–1261.4	899.1	7.77 (=0.24*b*^3^)	0.7–4.6	142.6 ± 3.4
4.8	22.8 ± 0.5	11.5–15.5	14.0	491.5–1187.1	867.6	7.40 (=0.23*b*^3^)	0.4–4.7	143.5 ± 3.3
9.6	23.2 ± 0.3	11.7–15.9	14.5	518.0–1277.5	967.3	8.47 (=0.26*b*^3^)	0.7–4.2	149.2 ± 5.9

^1^ For GaN single crystals, the magnitude of the Burgers vector **b** = 1/3[2−1−10] is *b* = 3.191 Å [27].

## Data Availability

Not applicable.
